# Korean Domestic Tourists’ Decision-Making Process under Threat of COVID-19

**DOI:** 10.3390/ijerph182010835

**Published:** 2021-10-15

**Authors:** JunHui Wang, Yunseon Choe, HakJun Song

**Affiliations:** 1 College of Tourism, Hunan Normal University, 36 Lushan Road, Yuelu District, Changsha 410081, China; junhuiw@hunnu.edu.cn; 2 School of Community Resources and Development, Watts College of Public Service and Community Solutions, Arizona State University, 411 N. Central Ave, Phoenix, AZ 85004, USA; Yunseon.Choe@asu.edu; 3 Department of Tourism Development and Management, The Hainan University-Arizona State University Joint International Tourism College, Hainan University, 58 Renmin Road, Haikou 570004, China; 4 College of Tourism and Fashion, Pai Chai University, 155-40 Baejae-ro, Seo-gu, Daejeon 302-735, Korea

**Keywords:** domestic travel, expectation, behavioral intention, mass media effect, policy

## Abstract

The purpose of this study is to build a theoretical framework to explain Korean domestic tourists’ decision-making process under COVID-19 by applying an extended model of goal-directed behavior. The role of positive expectation of COVID-19, the effect of mass media, and perception of government policy were considered as new variables in the process of tourism decision-making. The results of this present study show that positive and negative anticipated emotion, positive expectation, and the frequency of past behavior positively influence the desire for travel domestically within the next three months, while the effect of mass media negatively affected the desire for travel domestically within the next three months. Two anticipated emotions and positive expectations were positive antecedents of the desire for travel domestically within this year. The anticipated emotions and the effect of mass media affected the desire to travel domestically next year. The results of this tourist’s decision-making research will offer government, the tourism sector, and policy decision-makers better insights for establishing tourism policy responses and create safe destinations to help an adequate recovery and development of the tourism industry.

## 1. Introduction

In the past 40 years, the world has experienced many major epidemics/pandemics. However, none have had a similar impact on the global economy, such as Coronavirus disease 2019 (hereafter COVID-19) [1]. The COVID-19 worldwide confirmed cases to date (as of 24 September 2021) have exceeded 231 million, deaths have surpassed 4.74 million [2], and unemployment has increased sharply in many countries [3]. Since the first COVID-19 case was identified in South Korea (hereafter Korea), a subject of the current study, 298,402 COVID-19 cases, and 2441 deaths were recorded as of 25 September 2021 [4]. It has impacted every aspect of individual life and society in Korea. In the early stages, to prevent the widespread of COVID-19, the Korean government implemented not only non-pharmaceutical interventions (e.g., social distancing, wearing a mask) but also prompt, extensive tests for COVID-19, including drive-through testing centers for COVID-19 [5]. However, due to the impacts of COVID-19, Korea’s unemployment rate increased to 5.4% a 21-year high in January 2021 [6]; social isolation creates feelings of fear and anxiety, along with the sense of isolation, confusion, and fatigue; behavioral disposition decreases contact between persons switching to online purchases/lectures, self-service counters, video-conferences, and virtual events [7].

Korea is one of the countries that has responded well to COVID-19, domestic tourism in Korea has recovered more quickly since global travel restrictions cause a domestic tourism boom [8]. Even during COVID-19, many Koreans have begun to seek out travel activities and opportunities that are particularly suited to fulfill their travel needs while minimizing the risk of exposure to COVID-19 [9]. For travel in Korea, tourists need to follow practical guidelines for health and safe travel instructions from national health authorities based on in-travel social distancing, including having a limited number of visitors per day, offering low-touch or private amenities, and participating in low-risk outdoor activities. Popular tourist destinations, national parks, and ski resorts were shut down nationwide starting from Christmas Eve to New Year’s Day [10]. Particularly, COVID-19 has reshaped Koreans’ travel patterns with more tourists engaging in contactless and spontaneous travel to escape into nature, going to camping sites or luxury hotels reserved for family, taking a road trip, or traveling alone rather than visiting crowed areas as planning ahead is difficult because of the uncertainty surrounding the COVID-19 situation [8,11]. As a result, demand for campsites increased by 73% from February to April in 2020 compared to the same period in 2019 [12]. Many luxury hotels offer reserved facilities (i.e., private pool) and services (i.e., private dining rooms, buffet room service, exclusive private lounges, live chat concierge service). Along with the domestic tourism boom in Korea, local governments in tourism have promoted hiking trails, nature-based tourism destinations (i.e., parks, forests), and scenic driving routes for contactless tourism destinations to fulfill the needs for tourists to escape into nature [9]. Under this uncertain tourism phenomenon, understanding travelers’ behavior intention is critical for destination management organizations, practitioners, and researchers in tourism [13]. Tourists’ behavioral intention under risky situations that can cause physical harm may have a significant impact on travel decision-making [14]. Tourists’ behavioral intention is an inherently multifaceted and complex decision-making process of the tourist, and derived intention plays a crucial role in leading to actual behavior [15,16]. Lam and Hsu emphasized that a tourist’s behavioral intention to visit a tourism destination is helpful in defining one’s decision-making process [17]. Hence, as a more advanced model is needed to improve the capabilities of prior tourist behavior theories [18], this study attempts to build a theoretical framework that explains Korean domestic tourists’ decision-making process considering variables (i.e., attitudes, subjective norms, perceived behavioral control) under COVID-19. The new theoretical framework from this research was derived from an extended model of goal-directed behavior (EMGB) as it has strong predictive power for a wide range of tourist behavior that helps researchers and practitioners to better understand tourists’ decision-making processes [19].

This study thus highlights the behavioral intention among Korean domestic travelers during COVID since no research has yet investigated Korean domestic tourists’ decision-making process under threat of COVID-19. Despite many researchers in tourism agreeing that examining factors that lead to travelers’ behavioral intentions offer significant knowledge in the process of tourism decision-making, most research conducted on the impact of COVID-19 on tourism has only been qualitative research to explore new phenomena [20] focusing on crisis management, sustainability development, and tourism destination image [1,21,22,23,24,25,26]. In this situation, the purpose of this study was to build a theoretical framework to explain Korean domestic tourists’ decision-making process under COVID-19 by applying the EMGB model. In particular, this research aims (1) to understand the role of positive expectation of COVID-19, the effect of mass media and perception of government policy in forming domestic tourists’ desire, and behavioral intention; (2) to identify key factors that affect domestic tourists’ behavioral intention; and (3) to provide the basis for government and tourism operators to recover from the tourism crisis. The findings of this study on tourists’ decision-making process under COVID-19 can offer a better understanding of tourists’ behavioral intentions when establishing tourism policy responses and creating safe destinations to help with adequate recovery and development of the tourism industry. The findings of this study will provide not only theoretical value in the examination of behavior intention of domestic tourists, but also a practical application for understanding the decision-making process for travel under COVID-19.

## 2. Literature Review

### 2.1. Model of Goal-Directed Behavior (MGB)

In this research, the model of goal-directed behavior (MGB) was employed as a main theoretical framework to achieve the research goal. Perugini and Bagozzi [18] proposed a more advanced model called the goal-directed behavior model (MGB) in order to improve the capabilities of previous tourist behavior theories. MGB integrates four structures (i.e., anticipated positive emotion, anticipated negative emotion, desire, and past behavior). Specifically, all related variables (e.g., attitudes, subjective norms, perceived behavior control, and anticipate emotions) will indirectly affect behavioral intentions through desire. It is worth noting that perceived behavior control and past behavior can directly affect behavior. According to Ajzen [27], attitude means the degree of an individual’s evaluation or evaluation of a certain behavior; subjective norm is related to the perceived social pressure of performing or not performing behavior; perceived behavior control is the perceived difficulty of performing the behavior.

In terms of frequency of past behavior, it means that the behavior was performed over a relatively long period of time (usually 1 year) [18]. The role of anticipated emotions in MGB is related to the emotion caused when people consider the consequences of both goal success and failure [28]. Perugini and Bagozzi argued that social psychology models should include motivational and emotional processes that allow for a more concrete understanding of human behavior [18]. In addition, for the uncertain future, people may have forward-looking emotions about future behavior. Desire is also an important construct to understand human behavior because it is human nature to satisfy their desires. Desire refers to a state of mind that is related to a sense of longing for a person or object or a hope for results [29]. Desire is a state of mind generated by a continuous motivation process for specific behavior. However, due to the progress of pandemic conditions in Korea, this study divided desire into three stages according to time (i.e., desire of the next three months; desire of this year; desire of next year). Thus, hypotheses related to the MGB model should follow accordingly:

**Hypothesis** **1** **(H1).** *Attitude (ATT) has a positive influence on desire*.

**Hypothesis** **1** **(H1a).** *ATT has a positive influence on desire of next three month*.

**Hypothesis** **1** **(H1b).** *ATT has a positive influence on desire of this year*.

**Hypothesis** **1** **(H1c).** *ATT has a positive influence on desire of next year*.

**Hypothesis** **2** **(H2).** *Subjective norm (SN) has a positive influence on desire*.

**Hypothesis** **2** **(H2a).** *SN has a positive influence on desire of next three month*.

**Hypothesis** **2** **(H2b).** *SN has a positive influence on desire of this year*.

**Hypothesis** **2** **(H2c).** *SN has a positive influence on desire of next year*.

**Hypothesis** **3** **(H3).** *Positive anticipated emotion (PAE) has a positive influence on desire*.

**Hypothesis** **3** **(H3a).** *PAE has a positive influence on desire of next three month*.

**Hypothesis** **3** **(H3b).** *PAE has a positive influence on desire of this year*.

**Hypothesis** **3** **(H3c).** *PNE has a positive influence on desire of next year*.

**Hypothesis** **4** **(H4).** *Negative anticipated emotion (NAE) has a positive influence on desire*.

**Hypothesis** **4** **(H4a).** *NAE has a positive influence on desire of next three month*.

**Hypothesis** **4** **(H4b).** *NAE has a positive influence on desire of this year*.

**Hypothesis** **4** **(H4c).** *NAE has a positive influence on desire of next year*.

**Hypothesis** **5** **(H5).** *Perceived behavioral control (PBC) has a positive influence on desire*.

**Hypothesis** **5** **(H5a).** *PBC has a positive influence on desire of next three month*.

**Hypothesis** **5** **(H5b).** *PBC has a positive influence on desire of this year*.

**Hypothesis** **5** **(H5c).** *PBC has a positive influence on desire of next year*.

**Hypothesis** **6** **(H6).** *Frequency of past behavior (FPB) has a positive influence on desire and behavioral intention*.

**Hypothesis** **6** **(H6a).** *FPB has a positive influence on desire of next three month*.

**Hypothesis** **6** **(H6b).** *FPB has a positive influence on desire of this year*.

**Hypothesis** **6** **(H6c).** *FPB has a positive influence on desire of next year*.

**Hypothesis** **6** **(H6d).** *FPB has a positive influence on behavioral intention*.

MGB has been proven to be an advanced model that can understand tourists’ behavior in a wider range. However, in certain situations or contexts, it may be necessary to consider some relevant variables to explain and predict behavioral intention and actual behavior in the MGB [18]. Based on these criteria, positive expectation of COVID-19, the effect of mass media, and perception of government policy were integrated as new constructs to extend the original MGB in this study since they are potentially appropriate to specific behaviors under COVID-19. Specifically, expectation plays an important role in tourists’ decision-making [30,31], the effect of mass media plays an important role in tourists’ decision-making [19], the perception of government policy also plays an important role in tourists’ decision-making [32].

### 2.2. Expectation

Higgs, Polonsky, and Hollick [33] pointed out that expectations usually refer to the predictions of the expected results or performance of future products or services in the minds of consumers. Feather [34] argued that what a person does in a situation is related to the subjective value of the expectations held by that person and the consequences that can occur after actions. In this regard, people have always believed that expectations are important in explaining individual behavior, especially their economic behavior [35]. In the marketing literature, expectations have been regarded as the benchmark used by consumers to determine product or service satisfaction or evaluate performance [36]. Tourists usually have initial expectations for the product or service before using the product or service. These expectations are formed through the information in advertisements and the reputation of other consumers in the past [37]. Del Bosque et al. [38] showed that expectations were formed by past experience, tourists’ previous satisfaction with services, communication between service providers (such as promises), and tourists’ perception of services.

### 2.3. Mass Media Effect

Mass media has been attributed as having considerable power in shaping opinions and beliefs, changing habits of life, and actively molding behavior [39,40]. The public is likely to be affected by what mass media delivers. Mass media is also a channel for persuasion and mobilization. Due to the continuous and selective interaction between the self and the media, media plays a certain role in shaping individual behavior and self-concept [40]. Thus the information conveyed by the media will produce an intentional or unintentional influence on people’s views [41]. In addition, mass media not only delivers factual information about a certain issue but also arouses interest in the topic by emphasizing the topic and encouraging audiences to pay attention. With the increase in mass media coverage, it is plausible that the public’s attention to COVID-19 has increased dramatically. Mass media may increase or decrease the public’s anxiety about the status of the pandemic and increase or decrease the public’s confidence in the stability of the pandemic. As far as domestic tourists are concerned, mass media can have an impact on tourists’ perception of the safety of travel destinations and their travel intentions.

#### 2.3.1. Relationship between Mass Media Effect, Positive Expectation, and Desire

Media is an important channel for the public to obtain information about particular issues, and it is also an influential tool to arouse public perception and awareness of this issue [39,42,43]. Hansen [44] stated that the media tends to strongly influence the public’s priority in matters of concern as the public usually judges which topic is important based on the emphasis of a topic. Social scientists have extensively discussed the influence of the media on public perceptions, attitudes, and behaviors [44].

The development of the internet and media is the main factor for changes in the tourism industry and tourist purchase and experience [45]. Tasci and Boylu found that mass media coverage continues to increase the impact of disaster events on local tourism [46]. Reisinger and Mavondo [47] proposed that once a tourist realizes the potential danger or risk in a tourist destination, his/her willingness to visit decreases. The spread of mass media information can also have a positive or negative impact on positive expectations. For example, mass media information related to the progress of vaccine research may increase confidence in defeating the epidemic or may cause frustration at the failure of vaccine development. As the spread of information has likely accelerated the COVID-19 network and media [48], visitors will naturally be aware of the dangers of the external environment when making tourism decisions. Thus, it would be hypothesized that the mass media effect has a positive effect on positive expectations of COVID and desire.

**Hypothesis** **7** **(H7).** *Mass media effect (MME) has a positive effect on positive expectation of COVID-19 (PEC) and desire*.

**Hypothesis** **7** **(H7a).** *MME has a positive influence on desire of next three month*.

**Hypothesis** **7** **(H7b).** *MME has a positive influence on desire of this year*.

**Hypothesis** **7** **(H7c).** *MME has a positive influence on desire of next year*.

**Hypothesis** **7** **(H7d).** *MME has a positive influence on PEC*.

#### 2.3.2. Relationship between Perception of Government Policy, Positive Expectation, and Desire

In the early-stage trials for the COVID-19 vaccine and with the limited available medical interventions, most countries adopted various forms of non-pharmaceutical interventions (NPI) such as national lockdown (including stay-at-home order implemented in the U.S.), social isolation, closing schools, and non-essential businesses, canceling or postponing events (i.e., conferences, trade shows, concerts, festivals, political debates, in-person elections, sports seasons, and Olympics). In this situation, policies may have a significant impact on tourists’ psychology. The implementation of policies to deal with COVID-19 can psychologically reduce the public’s internal insecurity, general anxiety, and anxiety caused by COVID-19. From a practical point of view, the implementation of strong policy measures related to COVID-19 can also create a healthier domestic living environment with a lower degree of danger for the public.

The improvement of the domestic epidemic is likely to help the recovery and development of the tourism industry. In addition, the transparency around government policies and information helps tourists who desire domestic travel to obtain information faster and more effectively, thus as to avoid risks as much as possible and protect themselves. Government policies will have an impact on the personal behavior of tourists. However, limited research has focused on the impact of government policy on individual behavior. For instance, Wang et al. [32] investigated the effect of Korean government policy on smog to better capture the formation process of tourists’ behavioral intentions for domestic travel by applying the protection motivation theory. The authors concluded that government policy on smog has a positive indirect effect on the behavioral intentions of tourists. In addition, Lee, Jung, and Kim [49] investigated the effect of a policy intervention on users’ intention to change handsets and expenses on mobile communications. They found that policy intervention on handset subsidies lowered users’ willingness to switch handsets and increased spending on expenses in handset installment. This results in the following hypothesis:

**Hypothesis** **8** **(H8).** *Perception of government policy (PLY) has a positive effect on positive expectation of COVID-19 (PEC) and desire*.

**Hypothesis** **8** **(H8a).** *PLY has a positive influence on desire of next three month*.

**Hypothesis** **8** **(H8b).** *PLY has a positive influence on desire of this year*.

**Hypothesis** **8** **(H8c).** *PLY has a positive influence on desire of next year*.

**Hypothesis** **8** **(H8d).** *PLY has a positive influence on PEC*.

#### 2.3.3. Relationship between Positive Expectation of COVID-19 and Desire and Intention

**Figure 1:**
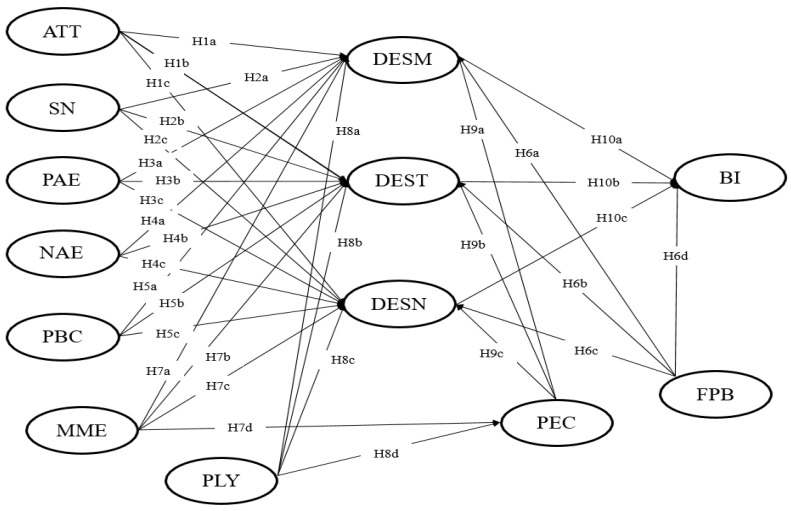
Research model.

According to Crompton and Ankomah [50], expectation, as a cognitive process, particularly influences evaluation and decision-making process. Song et al. [30] verified the expectation of tourist visa exemption is a cognitive anticipation of benefits that stimulates tourists’ desire and travel intentions. They explained that the expectation of Chinese tourists gaining visa-free entry can effectively stimulate their strong desire to travel to Korea and enhance their behavioral intentions. Song et al. [31] believe that casino employees’ expectations of responsible gambling strategies have inspired their attitudes towards casino gamblers because employees believe that the company’s responsible gambling strategies help minimize the negative impact on customers. Therefore, it can be assumed that positive expectations of COVID-19 will have a positive effect on desire. The research model of the current study related to the hypotheses presented earlier is shown in Figure 1.

**Hypothesis** **9** **(H9).** *Positive expectation of COVID-19 (PEC) has a positive effect on desire*.

**Hypothesis** **9** **(H9a).** *PEC has a positive influence on desire of next three month*.

**Hypothesis** **9** **(H9b).** *PEC has a positive influence on desire of this year*.

**Hypothesis** **9** **(H9c).** *PEC has a positive influence on desire of next year*.

**Hypothesis** **10** **(H10).** *Desire has a positive influence on behavioral intention*.

**Hypothesis** **10** **(H10a).** *DESM has a positive influence on behavioral intention*.

**Hypothesis** **10** **(H10b).** *DEST has a positive influence on behavioral intention*.

**Hypothesis** **10** **(H10c).** *DESN has a positive influence on behavioral intention*.

## 3. Method

### 3.1. Variable Measurement

To develop the questionnaire items for this study, a preliminary list of measurement items was created after a literature review. In addition, in accordance with the argument of Kline [51], this study attempted to develop a questionnaire item using multiple indicators to increase the efficiency of the structure. All factors were tested by a 5-point Likert scale ranging from strongly disagree (1) to strongly agree (5). In detail, the subjects’ attitudes with 4 items (e.g., “I think domestic travel is positive”), the subjective norm with four items (e.g., “Those who influence my decision will approve of my domestic travel”), and perceived behavioral control with three items (e.g., “I can travel domestically at any time I want”) based on previous research [27,30,52,53,54]. Anticipated emotions were operationalized by 8 items (4 items for anticipated positive emotions and 4 items for anticipated negative emotions) (e.g., “I will be excited if I can travel domestically”, “I will be angry if I can’t travel domestically”) based on previous research [18,30,53]. The mass media effect was operationalized by 4 items (e.g., “Mass media (TV, news, internet) notifies of the risk of COVID-19”) based on Kim and Kim [55]’s research. The perception of government policy against COVID-19 was operationalized by four items (e.g., “The government’s policies against COVID-19 are stable”) based on Wang et al. [32]’s research. The positive expectation of COVID-19 with 4 items (e.g., “I am optimistic about the future of COVID-19”), desire to travel domestically with 9 items (3 items for the next 3 months, 3 items for this year and 3 items for next year) and behavioral intention with 4 items was based on previous research [16,18,56]. The frequency of past behavior was assessed with a single item (i.e., how many times have you traveled domestically in the last year?) based on previous studies [57,58]. Demographic questions of respondents (i.e., gender, age, education level, marriage status, occupation, and income level) were included in the last section of the questionnaire to gather the sample characteristics. The questionnaire was originally written in Korean and then translated into English by tourism scholars and professional translators to ensure the accuracy of the translation and avoid construction errors.

### 3.2. Data Collection

In this study, an online panel survey was conducted with the assistance of Embrain, which is the largest online research firm in Korea with 6.4 million panelists. The online survey was administered from 26 May to 2 June 2020, implementing a quota sampling taking into account the structure by age, gender, and regions of the census data [6]. The online research firm sent a survey to a targeted population of respondents and in total 711 questionnaires were used in data analysis. Gay and Airasain [59] pointed out that if the population size is around 5000 or more, the structural equation modeling (SEM) sample size should exceed 400. Therefore, the sample size of the SEM in the current study was sufficient to maintain the accuracy of the estimation. In order to analyze the hypothetical structural model, R-studio statistical package was used to analyze the collected data.

## 4. Results

### 4.1. Demographic Characteristics of Samples

The demographic characteristics of the respondents are shown in Table 1. The proportion of male respondents was 50.2% and that of the female 49.8%. The majority of respondents were ages 50–59 (27.9%) and ages 40–49 (27.6%). More than half of the respondents held a university degree (59.8%). Most respondents were married (56.7%). The two most common occupations in the sample were office staff (36.4%) and experts or technicians (14.9%). A total of 25.7% of respondents’ monthly income ranged from KRW 2–2.9 million and 18.3% between KRW 3–3.9 million.

### 4.2. Measurement Model

In the first step, the measurement model for the EMGB variables was estimated by performing a Confirmatory Factor Analysis [60]. The proposed measurement model fit the data well with the good-fit shown in Table 2 (CFI = 0.954, NFI = 0.918, NNFI = 0.948, RMSEA = 0.040). All factor loadings were greater than the minimum criteria of 0.5 with a significantly associated t-value, supporting the convergent validity of the measurement model for the EMGB [60,61]. In terms of reliability, each construct had a sufficient level of reliability because the values of Cronbach’s alpha ranged from 0.828 to 0.959 (shown in Table 3), exceeding the suggested minimum criteria of 0.7 [62].

Convergent and discriminant validity was checked to judge construct validity. As shown in Table 4, all average variance extracted (AVE) and composite reliability values for the multi-item scales were greater than the minimum criteria of 0.5 and 0.7, respectively [61]. The results indicate that the measurement model had a sufficient level of convergent validity. In addition, all AVEs of each construct were greater than the squared correlation in Table 4, which demonstrates satisfactory discriminant validity.

### 4.3. Structural Model

**Figure 2:**
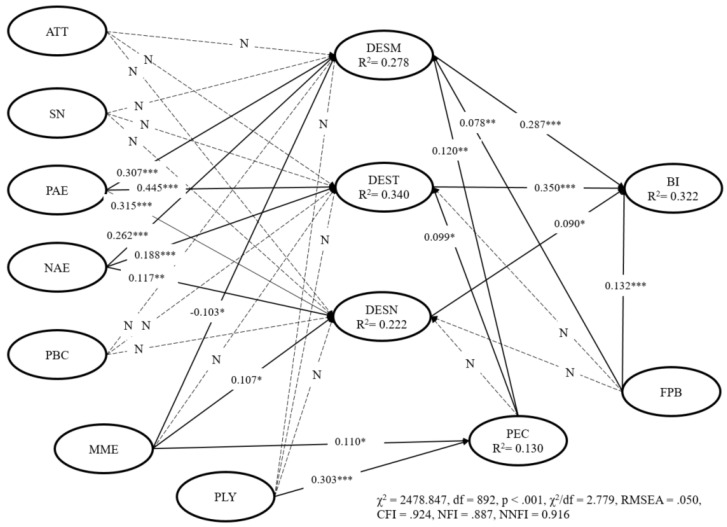
Results of the structural model. Notes: * *p* < 0.05, ** *p* < 0.01, *** *p* < 0.001.

The structural model of the EMGB was found to fit the data well with better goodness-of-fit (χ^2^ = 2484.847, df = 892, χ^2^/df = 2.78 < 3, CFI = 0.924, NFI = 0.887, NNFI = 0.916, RMSEA = 0.050). The results of the EMGB are shown in Table 5 and Figure 2. Four predictor variables (positive anticipated emotion (βPAE → DESM = 0.307, t = 4.993, *p* < 0.001), negative anticipated emotion (βNAE → DESM = 0.262, t = 6.273, *p* < 0.001), positive expectation (βPEC → DESM = 0.120, t = 2.740, *p* < 0.01), the frequency of past behavior (βFPB → DESM = 0.078, t = 3.108, *p* < 0.01)) positively impacted the desire of travel domestically within the next three months; while effect of mass media (βMME → DESM = −0.103, t = −2.446, *p* < 0.05) negatively impacted the desire of travel domestically within the next three months, supporting H3a, H4a, H6a, H7a, and H9a. However, attitude (t = −0.713, *p* > 0.05, not significant), subject norm (t = 0.815, *p* > 0.05, not significant), and perceived behavioral control (t = 1.279, *p* > 0.05, not significant), policy (t = 1.735, *p* > 0.05, not significant) were not statistically significant to predict desire of travel domestically within the next three months, rejecting H1a, H2a, H5a, H8a. Only positive anticipated emotion (βPAE → DEST = 0.445, t = 7.391, *p* < 0.001), negative anticipated emotion (βNAE → DEST = 0.188, t = 4.972, *p* < 0.001), and positive expectation (βPEC → DEST = 0.099, t = 2.378, *p* < 0.05) positively impacted the desire of travel domestically within this year, supporting H3b, H4b and H9b. Only positive anticipated emotion (βPAE → DESN = 0.315, t = 4.765, *p* < 0.001), negative anticipated emotion (βNAE → DESN = 0.117, t = 2.821, *p* < 0.01), and effect of mass media (βMME → DESN = 0.107, t = 2.080, *p* < 0.05) positively impacted the desire of travel domestically next year, supporting H3c, H4c, and H7c.

The relationships between mass media effect, policy, and positive expectation were also found to be positive and significant (βMME → PEC = 0.112, t = 2.290, *p* < 0.05; βPLY → PEC = 0.303, t = 6.711, *p* < 0.001), supporting H6d, H7d, H8d. In addition, the relationships between desire (desire of next three months, desire of this year, desire of next year), frequency of past behavior and behavioral intention were found to be positive and significant (βDESM → BI = 0.287, t = 7.859, *p* < 0.001; βDESN → BI = 0.350, t = 11.199, *p* < 0.001; βDESN → BI = 0.090, t = 2.526, *p* < 0.05; βFPB → BI = 0.132, t = 3.541, *p* < 0.001), supporting H10a, H10b, H10c, H6d.

## 5. Conclusions

### 5.1. Theoretical Implications

Although tourism has a highly vulnerable resiliency to the external crisis by tourists, tourist destinations and residents living in tourism areas can help to mitigate vulnerabilities [63]. It is clear that current crisis impacts from COVID-19 were most significant at the local or tourism destination levels, such as tour cancellations, facility closures, changes in air and cruise routes, and loss of access to tourism destinations [63,64]. In this situation, the EMGB in this study was successfully broadened by incorporating the tourists’ decision-making process under COVID-19 with certain theoretical implications. This study found the decision-making process to travel under COVID-19 is complicated and made carefully from various viewpoints. The results of this study showed that the MGB is a useful approach to describe the travel decision-making process under COVID-19 by adding related factors (e.g., positive expectations, mass media effect, and perception of government policy), dividing desire into the desire of next three months/this year/next year. In all antecedent variables of the EMGB, desire of this year was found to be the most important latent variable for behavioral intention and it also acted as a sufficient impetus for behavioral intention formation. In the result of current study, anticipated positive emotion and anticipated negative emotion were the most important determinants of desire, no matter the desire of the next three months or this year or next year, compared to other variables. This finding revealed that domestic tourists were likely to travel domestically under COVID-19 due to emotional factors rather than other cognitive factors. This can be explained by tourism experiences often including satisfying and pleasurable emotions [65,66], which would not be discovered when employing the TPB. Moreover, the frequency of past behavior only significantly impacts the desire for the next three months.

An interesting result was that there was a negative significant causal relationship between mass media effect and desire of next three months, no significant causal relationship between mass media effect and desire of this year, while there was a positive significant causal relationship between mass media effect and desire of next year. The reason may be that the severity of the epidemic has been widely reported and publicized by the mass media in the early stage, which caused certain psychological pressures on the public and thus affected their travel intentions. However, with the passage of time, people’s sensitivity to the epidemic decreased, and the influence of the media also became less. With the progress of vaccine research, more media began to promote positive information, which will gradually increase people’s confidence in overcoming the epidemic and promote tourists’ tourism activities. Frequency of past behavior can have a positive influence on behavioral intention directly or through desire (specifically desire for travel domestically within the next three months). This implies that desire is likely to perform as a sufficient impetus for behavioral intention formation. As expected, desire is a strong mediated variable between the endmost constructs of the model and behavioral intention. Interestingly, perception of government policy and mass media effect has been shown to have a significant impact on positive expectations for COVID-19. The former was more powerful than the latter. Perceptions of government policy were not statistically significant directly on desire but indirectly significant through positive expectations for COVID-19. Moreover, the positive expectation for COVID-19 was found to have a great influence on the desire for the next three months and the desire for this year. On the other hand, it did not significantly affect the desire for next year. It indicates that positive expectations of the epidemic can affect travel desires in the next three months and this year, but not next year. This may be because psychological expectations are time-sensitive and can be used to predict and estimate tourists’ desires and behavior in the relatively short term. Due to changes in the epidemic situation, the situation next year cannot be estimated yet. Thus the impact is estimated as not significant.

### 5.2. Practical Implications

Practical implications are provided by analysis findings. As revealed in the study that mass media can strengthen the perception of COVID-19 of tourists, Korean government needs to consider the influence of mass media. In an early stage of the outbreak, supervision and management work should be conducted to ensure the accuracy and effectiveness of mass media information, particularly, penalties on and management of social media around false information and rumor, which may lead to intentional social panic. Dissemination of positive information and news can increase public confidence and courage in responding to the epidemic. As far as government policy is concerned, open and transparent policy measures are necessary. In the current epidemic prevention and control work, it is particularly important to ensure the timely disclosure of the route information of infected persons. Government should continue to close crowd-intensive entertainment venues and encourage people to relax at home or go to areas where people are less likely to congregate and ensure that those who return home or visit Korea implement self-isolation to cut off the overseas transmission of the epidemic. Under conditions where domestic epidemics tend to stabilize, domestic travel can be encouraged to restore the domestic economy. Promoting government policies through mass media increased people’s confidence in the government’s ability to overcome the epidemic. Both mass media and government policies have a positive impact on individuals’ positive expectations. This means that the government has taken active and effective measures to deal with the epidemic, vigorously supported and promoted the research and development of vaccines, helped unemployment caused by the epidemic, and so on. With the positive reports by mass media, these measures have alleviated people’s panic and anxiety and enhanced confidence.

Under the current pandemic conditions, whether it is the next three months, this year, or next year, the only important factor affecting the desire to travel is the anticipated emotion. Both positive and negative anticipated emotions indicate that the only prerequisite for influencing tourists’ desire to travel is anticipated emotion, which is not related to attitudes, subject norms, or perceived behavioral control. It also means that attractive tourist destinations or products that stimulate the inner needs of tourists are the keys to success. Thus, providing personalized or customized travel products or services to attract tourists’ attention is very important to marketers. Since pandemic prevention and control is not over, the use of advanced AI to provide intelligent high-tech services is also of great significance. Last but not least, ensuring a focus on safety in current product development or design is of vital importance for the restoration of the tourism industry.

### 5.3. Limitations and Future Research

This study was conducted during COVID-19. Evidently, tourists’ decision-making process and travel intention have been and will be influenced by the current situation in Korea. Furthermore, there were differences in the regional distribution of COVID-19, and tourists in different regions have different perceptions of COVID-19. It is vital to focus on investigating the travel intention of the people of a destination region. Thus, the findings of this study cannot be generalized but rather can be used to provide a rich, contextualized understanding of Korean domestic tourists’ decision-making process under COVID-19, requiring careful interpretation of the results. If the further outbreak of the pandemic continues in Korea, financial markets, the global economy, and the consumer confidence index may decline. In future research, further investigation is needed on how the outbreak of the pandemic impacts the recovery of domestic and international tourism, when will the tourism industry recover from COVID-19, and whether and how the tourist’s decision-making process will change after the pandemic. In addition, the statistical results show that the explanatory power of this article is only about 30%. The reason may be the incomplete preparation of the test work in the early stage of the questionnaire design, resulting in a slightly insufficient explanatory power of the data results. Future research could consider replacing other factors with a better fit to carry out research to achieve a better interpretation of the tourist behavior decision process under COVID-19.

## Figures and Tables

**Table 1:** Demographic characteristics of the respondents (N = 711).

Characteristic	N (%)	Characteristic	N (%)
Gender		Marital status	
Male	357 (50.2)	Single	298 (41.9)
Female	354 (49.8)	Married	403 (56.7)
		Other	10 (1.4)
Education level		Age	
High school or lesser level	103 (14.5)	20–29 years old	152 (21.3)
College level	111 (15.6)	30–39 years old	165 (23.2)
Undergraduate level	425 (59.8)	40–49 years old	196 (27.6)
Graduate level	72 (10.1)	50–59 years old	198 (27.9)
Monthly income (KRW) *		Occupation	
Less than 1 million	108 (15.2)	Technician	106 (14.9)
1–1.99 million	81 (11.4)	Businessman	47 (6.6)
2–2.99 million	183 (25.7)	Sales and service employee	47 (6.6)
3–3.99 million	130 (18.3)	Office worker	259 (36.4)
4–4.99 million	83 (11.7)	Government employee	30 (4.2)
5–5.99 million	70 (9.9)	Student	58 (8.2)
6–6.99 million	23 (3.2)	Homemaker	87 (12.2)
7–7.99 million	16 (2.2)	Freelance	48 (6.8)
Over 8 million	17 (2.4)	Retired	4 (0.6)
		Others	25 (3.5)

Note: * 1 U.S. Dollar is equivalent to 1165.41 Korean Won (KRW).

**Table 2:** Results of Goodness-of-fit indices for measurement model of EMGB.

Structural Model	χ^2^	df	Normed χ^2^	CFI	NFI	NNFI	RMSEA
Fit	1774.167	836	2.122	0.954	0.918	0.948	0.04
Suggested value *			≤3	≥0.9	≥0.9	≥0.9	≤0.08

Note: * Suggested values were based on Hair et al. [61].

**Table 3:** Reliability and confirmatory factor analysis.

Factors and Scale Items	Standardized Loading	Cronbach’sAlpha
F1: Attitude (ATT)		
I think domestic travel is positive.	0.879	0.933
I think domestic travel is beneficial.	0.905	
I think domestic travel is valuable.	0.881	
I think domestic travel is attractive.	0.863	
F2: Subjective Norm (SN)		
Those who influence my decision will approve of my domestic travel.	0.914	0.933
Those who influence my decision will support my domestic travel.	0.915	
Those who influence my decision will understand my domestic travel.	0.865	
Those who influence my decision will recommend domestic travel to me.	0.844	
F3: Perceived Behavioral Control (PBC)		
I can travel domestically at any time I want.	0.632	0.828
I have the overall ability to travel domestically.	0.941	
I have enough financial resources to travel domestically.	0.822	
F4: Positive Anticipated Emotion (PAE)		
I will be excited if I can travel domestically.	0.915	0.949
I will be glad if I can travel domestically.	0.909	
I will be satisfied if I can travel domestically.	0.894	
I will be happy if I can travel domestically.	0.915	
F5: Negative Anticipated Emotion (NAE)		
I will be angry if I can’t travel domestically.	0.896	0.928
I will be disappointed if I can’t travel domestically.	0.911	
I will be worried if I can’t travel domestically.	0.798	
I will be upset if I can’t travel domestically.	0.889	
F6: Mass Media Effect (MME)		
Mass media (TV, news, internet) notifies of the risk of COVID-19.	0.897	0.897
Mass media notifies of the severity of COVID-19.	0.902	
Mass media notifies of the negative impact of COVID-19 on human health.	0.863	
Mass media notifies of the negative impact of COVID-19 on modern society in Korea.	0.76	
F7: Policy (PLY)		
The government’s policies against COVID-19 are stable.	0.891	0.916
The government is trying to protect the people from COVID-19.	0.896	
The government’s policy is reliable	0.92	
The government is providing COVID-19 transparent information.	0.882	
F8: Positive Expectation (PEC)		
I am optimistic about the future of COVID-19.	0.856	0.942
I think COVID-19 will stabilize soon.	0.815	
I am optimistic about COVID-19 vaccine development.	0.752	
Despite many difficulties, I have an optimistic view of the stability of COVID-19.	0.895	
F9: Desire within the next three months (DESM)		
I want to travel domestically within the next three months	0.939	0.951
I hope to travel domestically within the next three months.	0.913	
I am eager to travel domestically within the next three months.	0.94	
F10: Desire within this year (DEST)		
I want to travel domestically within this year.	0.952	0.953
I hope to travel domestically within this year.	0.897	
I am eager to travel domestically within this year.	0.956	
F11: Desire of next year (DESN)		
I want to travel domestically next year.	0.946	0.959
I hope to travel domestically next year.	0.927	
I am eager to travel domestically next year.	0.952	
F12: Behavioral Intention (BI)		
I plan to travel domestically again in the near future.	0.688	0.887
I will make an effort to travel domestically in the near future.	0.81	
I have an intention to travel domestically again in the near future.	0.882	
I am willing to invest money and time to travel domestically in the near future.	0.883	

Notes: All standardized factors loadings are significant at *p* < 0.001.

**Table 4:** Results of measurement model (N = 711).

Construct	ATT	SN	PBC	PAE	NAE	PEC	MME	PLY	DESM	DEST	DESN	BI
ATT	0.778	0.559 (0.748)	0.173 (0.416)	0.540 (0.735)	0.090 (0.301)	0.062 (0.250)	0.124 (0.352)	0.115 (0.339)	0.115 (0.340)	0.153 (0.392)	0.120 (0.346)	0.423 (0.651)
SN	0.033	0.783	0.154 (0.392)	0.383 (0.619)	0.085 (0.292)	0.046 (0.215)	0.090 (0.301)	0.072 (0.269)	0.098 (0.314)	0.125 (0.353)	0.104 (0.323)	0.348 (0.590)
PBC	0.026	0.025	0.653	0.211 (0.460)	0.074 (0.273)	0.063 (0.251)	0.045 (0.211)	0.039 (0.198)	0.091 (0.302)	0.093 (0.304)	0.042 (0.206)	0.184 (0.429)
PAE	0.033	0.028	0.026	0.825	0.110 (0.331)	0.063 (0.252)	0.167 (0.408)	0.093 (0.304)	0.165 (0.406)	0.270 (0.519)	0.171 (0.413)	0.448 (0.669)
NAE	0.031	0.031	0.027	0.028	0.765	0.017 (0.129)	0.001 (0.023)	0.010 (0.102)	0.158 (0.397)	0.108 (0.328)	0.050 (0.224)	0.203 (0.450)
PEC	0.025	0.027	0.022	0.024	0.036	0.691	0.051 (0.227)	0.118 (0.344)	0.058 (0.242)	0.063 (0.251)	0.018 (0.134)	0.051 (0.225)
MME	0.024	0.022	0.020	0.024	0.028	0.026	0.735	0.157 (0.396)	0.007 (0.085)	0.083 (0.288)	0.075 (0.274)	0.058 (0.241)
PLY	0.026	0.023	0.020	0.025	0.032	0.030	0.029	0.805	0.039 (0.197)	0.057 (0.238)	0.053 (0.230)	0.067 (0.260)
DESM	0.031	0.032	0.028	0.030	0.040	0.037	0.028	0.032	0.866	0.493 * (0.702)	0.168 (0.410)	0.297 (0.545)
DEST	0.030	0.030	0.026	0.029	0.037	0.032	0.026	0.027	0.048	0.874	0.424 (0.651)	0.331 (0.576)
DESN	0.030	0.030	0.027	0.029	0.037	0.031	0.026	0.027	0.041	0.043	0.887	0.161 (0.401)
BI	0.033	0.030	0.026	0.029	0.035	0.027	0.023	0.023	0.036	0.034	0.031	0.672
CR	0.934	0.935	0.846	0.950	0.929	0.899	0.917	0.943	0.951	0.954	0.959	0.890

Note: ATT = Attitude; SN = Subjective Norm; PAE = Positive Anticipated Emotion; NAE = Negative Anticipated Emotion; PBC = Perceived Behavioral Control; PLY = Policy; PMS = Protection Motivation for Smog; BI = Behavioral Intention; CR = Composite Reliability; * = Highest correlation between pairs of constructs; The values of AVE are along the diagonal; Squared correlations among latent constructs are above the diagonal; Correlations among latent constructs are in the parentheses; Standard errors among latent constructs are below the diagonal.

**Table 5:** Standardized parameter estimates of structural model (N = 711).

Hypotheses		Coefficients	*t*-Values	Test of Hypotheses
H1a	ATT → DESM	−0.052	−0.713	Rejected
H2a	SN → DESM	0.050	0.815	Rejected
H3a	PAE → DESM	0.307 ***	4.993	Accepted
H4a	NAE → DESM	0.262 ***	6.273	Accepted
H5a	PBC → DESM	0.062	1.279	Rejected
H6a	FPB → DESM	0.078 **	3.103	Accepted
H7a	MME → DESM	−0.103 *	−2.446	Accepted
H8a	PLY → DESM	0.077	1.735	Rejected
H9a	PEC → DESM	0.120 **	2.740	Accepted
H1b	ATT → DEST	−0.096	−1.403	Rejected
H2b	SN → DEST	0.037	0.620	Rejected
H3b	PAE → DEST	0.445 ***	7.391	Accepted
H4b	NAE → DEST	0.188 ***	4.972	Accepted
H5b	PBC → DEST	0.027	0.627	Rejected
H6b	FPB → DEST	0.028	0.843	Rejected
H7b	MME → DEST	0.085	1.728	Rejected
H8b	PLY → DEST	0.043	1.002	Rejected
H9b	PEC → DEST	0.099 *	2.378	Accepted
H1c	ATT → DESN	−0.041	−0.613	Rejected
H2c	SN → DESN	0.085	1.467	Rejected
H3c	PAE → DESN	0.315 ***	4.765	Accepted
H4c	NAE → DESN	0.117 **	2.821	Accepted
H5c	PBC → DESN	−0.018	−0.385	Rejected
H6c	FPB → DESN	−0.013	−0.290	Rejected
H7c	MME → DESN	0.107 *	2.080	Accepted
H8c	PLY → DESN	0.079	1.880	Rejected
H9c	PEC → DESN	−0.006	−0.125	Rejected
H6d	FPB → BI	0.132 ***	3.541	Accepted
H7d	MME → PEC	0.110 *	2.290	Accepted
H8d	PLY → PEC	0.303 ***	6.771	Accepted
H10a	DESM → BI	0.287 ***	7.859	Accepted
H10b	DEST → BI	0.3500 ***	11.199	Accepted
H10c	DESN → BI	0.090 *	2.526	Accepted

Notes: * *p* < 0.05, ** *p* < 0.01, *** *p* < 0.001. ATT = Attitude; SN = Subjective Norm; PAE = Positive Anticipated Emotion; NAE = Negative Anticipated Emotion; PBC = Perceived Behavioral Control; MME = Mass Media Effect; PLY = Policy; PEC = Positive Expectation; DESM = Desire within the next three months; DEST = Desire within this year; DESN = Desire of next year; BI = Behavioral Intention.

## Data Availability

Not applicable.
